# Ischemic Stroke in a 28-Year-Old Young Adult Associated With Chronic Triptan and Non-Steroidal Anti-Inflammatory Drug (NSAID) Use

**DOI:** 10.7759/cureus.103037

**Published:** 2026-02-05

**Authors:** Natia Babukhadia, Tornike Jangirashvili, Lolita Shengelia

**Affiliations:** 1 Neurology, Tbilisi Central Hospital, Tbilisi, GEO; 2 Medicine and Surgery, Georgian-American University, Tbilisi, GEO; 3 Medical Education and Research, Georgian-American University, Tbilisi, GEO

**Keywords:** cortical infarction, : ischemic stroke, migraine in young adults, (nsaid) non-steroidal anti-inflammatory drugs, triptans

## Abstract

Ischemic stroke in young adults is an uncommon but clinically significant condition that frequently occurs in the absence of traditional cardiovascular risk factors. In this population, identification of nontraditional and potentially iatrogenic contributors is essential for accurate diagnosis and effective secondary prevention.

We report the case of a 28-year-old woman with normal body mass index who presented with acute-onset expressive aphasia accompanied by right-sided motor and sensory deficits. Her medical history was notable for chronic migraine treated with daily sumatriptan use over three years and intermittent administration of diclofenac (Fastenal), a non-selective NSAID, used as needed for pain control. A prior transient neurological episode three years earlier was associated with unremarkable neuroimaging. Current diagnostic evaluation with 1.5-Tesla brain magnetic resonance imaging (MRI), GE Brivo MR355 1.5T (GE Healthcare, Chicago, IL, USA), revealed two punctate acute ischemic lesions localized to the M4 cortical territory of the left middle cerebral artery. Laboratory investigations obtained on admission as part of the emergency department acute stroke protocol were largely unremarkable, except for subclinical hypothyroidism and borderline dyslipidemia.

This case underscores the diagnostic value of magnetic resonance imaging in identifying small cortical ischemic infarcts that may be undetectable on computed tomography. It also highlights the potential contributory role of chronic vasoconstrictive migraine therapies in conjunction with non-steroidal anti-inflammatory drug (NSAID) use and minor metabolic abnormalities in precipitating ischemic stroke in young adults. Increased clinical vigilance is warranted when evaluating young migraine patients presenting with acute focal neurological deficits.

## Introduction

This case underscores the diagnostic value of magnetic resonance imaging in identifying small cortical ischemic infarcts that may be undetectable on computed tomography. It also highlights the potential contributory role of chronic vasoconstrictive migraine therapies in conjunction with non-steroidal anti-inflammatory drug (NSAID) use and minor metabolic abnormalities in precipitating ischemic stroke in young adults. Increased clinical vigilance is warranted when evaluating young migraine patients presenting with acute focal neurological deficits.

Ischemic stroke remains a leading cause of morbidity and long-term disability worldwide; however, its occurrence in young adults represents a distinct clinical entity with heterogeneous etiologies and unique diagnostic challenges. Strokes in individuals under 50 years of age account for approximately 10-15% of all ischemic strokes and are frequently unrelated to traditional cardiovascular risk factors such as hypertension, diabetes mellitus, or advanced atherosclerosis [1,2]. Instead, this population often exhibits alternative mechanisms, including migraine-related vascular dysfunction, medication-induced vasoconstriction, metabolic disturbances, and prothrombotic states [3,4].

Migraine, particularly migraine with aura, has been increasingly recognized as an independent risk factor for ischemic stroke, especially in young women [5,6]. Recent studies have demonstrated that migraine may account for up to 35% of strokes in women under 35 years of age, making it one of the most important nontraditional risk factors in this population [7].

Proposed mechanisms include cortical spreading depolarization, endothelial dysfunction, platelet activation, and transient cerebral vasoconstriction [8,9]. Triptans, selective serotonin (5-HT1B/1D) receptor agonists widely used in migraine management, exert their therapeutic effect through cerebral vasoconstriction and inhibition of neurogenic inflammation [9]. While generally considered safe when used intermittently, chronic or excessive triptan exposure may potentiate vasospastic phenomena, particularly in susceptible individuals [10,11]. A recent Danish population-based study involving over 429,000 triptan users found that triptan initiation was associated with an increased risk of ischemic stroke, although the absolute risk remained very low at approximately 1 in 30,000 users [12].

NSAIDs have also been associated with increased cerebrovascular risk, potentially through effects on platelet aggregation, endothelial function, and blood pressure regulation [13-15]. In this case, the patient intermittently used ketoprofen lysine salt, a propionic acid-derived non-selective NSAID, which may contribute to cerebrovascular vulnerability when combined with other vasoconstrictive agents or subtle metabolic abnormalities [15].

Given the atypical risk profile in young stroke patients, advanced neuroimaging modalities such as magnetic resonance imaging play a pivotal role in detecting small-vessel or cortical infarctions that may be missed by conventional computed tomography [13,16]. This case report aims to illustrate the importance of comprehensive evaluation and heightened clinical suspicion in young adults presenting with acute neurological deficits, particularly in the context of chronic migraine therapy.

## Case presentation

A 28-year-old woman presented to the emergency department at Tbilisi Central Hospital with sudden-onset expressive aphasia accompanied by right-sided motor weakness and sensory deficits. On admission, neurological examination confirmed focal deficits involving speech production as well as right hemibody motor and sensory function. There was no impairment of consciousness. Her body mass index (BMI) was 23.1 kg/m², within the normal range.

Her medical history was notable for chronic migraine with aura, treated with daily sumatriptan use for approximately three years, along with intermittent use of ketoprofen lysine salt (Fastenal), a propionic acid-derived non-selective nonsteroidal anti-inflammatory drug. She denied combustible cigarette smoking, oral contraceptive use, illicit drug exposure, or known cardiovascular disease. The patient reported intermittent electronic cigarette use over an estimated two-year period, averaging approximately 15-20 puffs per day. This corresponds to an estimated exposure of roughly 5,500-7,300 puffs per year, equivalent to approximately nine to 12 standard disposable electronic cigarette devices annually (assuming ~600 puffs per device). A similar transient neurological episode had occurred three years earlier, at which time neuroimaging was reportedly unremarkable.

Initial diagnostic evaluation included brain magnetic resonance imaging performed according to an acute stroke protocol, incorporating T2-weighted turbo spin echo (T2-TSE), T2 fluid-attenuated inversion recovery (FLAIR), diffusion-weighted imaging (DWI; b = 1000), and three-dimensional time-of-flight (3D TOF) magnetic resonance angiography sequences.

**Figure 1:**
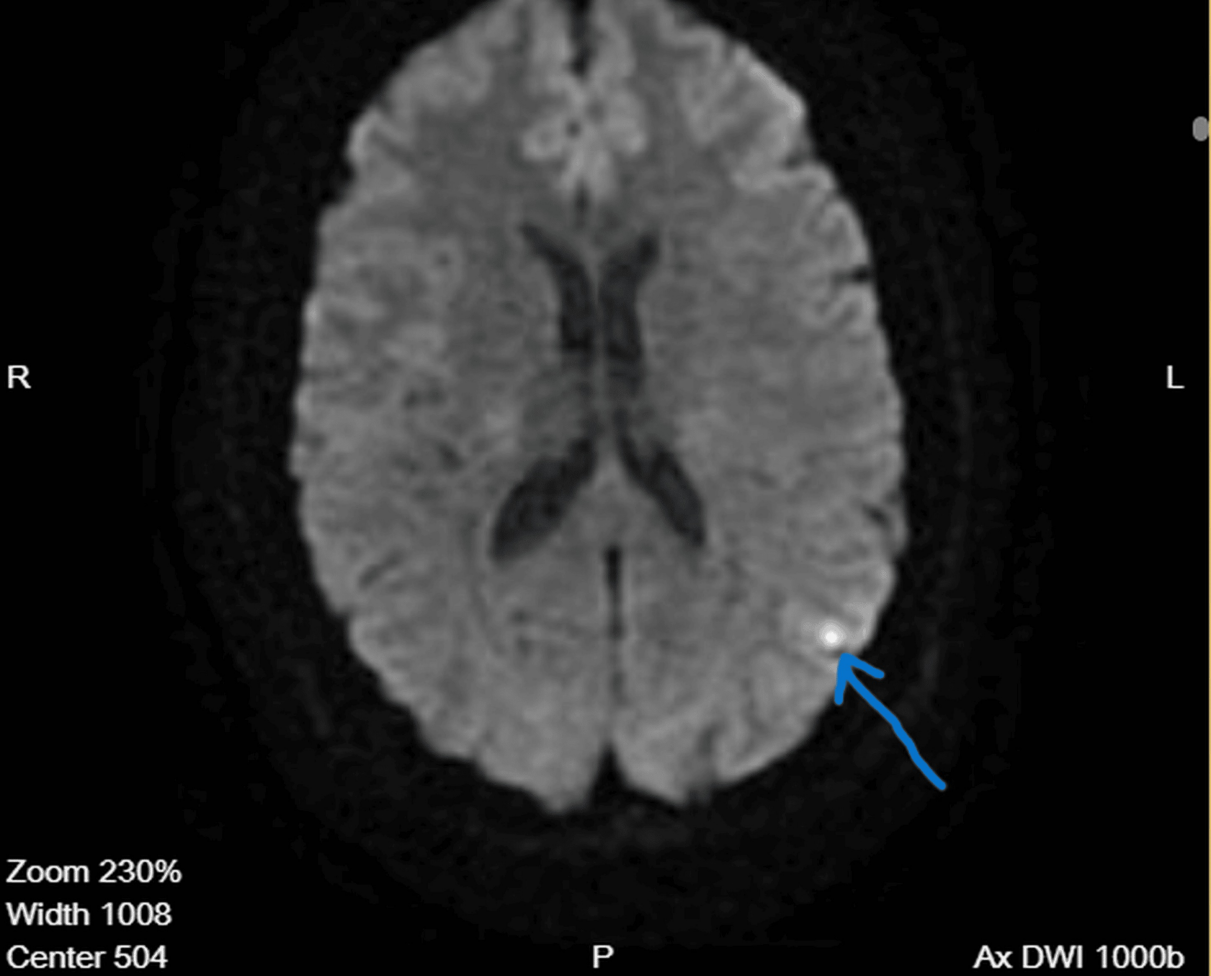
Diffusion-weighted MRI of the brain Diffusion-weighted magnetic resonance imaging (DWI) demonstrates a small punctate cortical focus of restricted diffusion in the left parietal lobe, corresponding to the distal M4 territory of the left middle cerebral artery. The arrow indicates the area of acute ischemic infarction.

**Figure 2:**
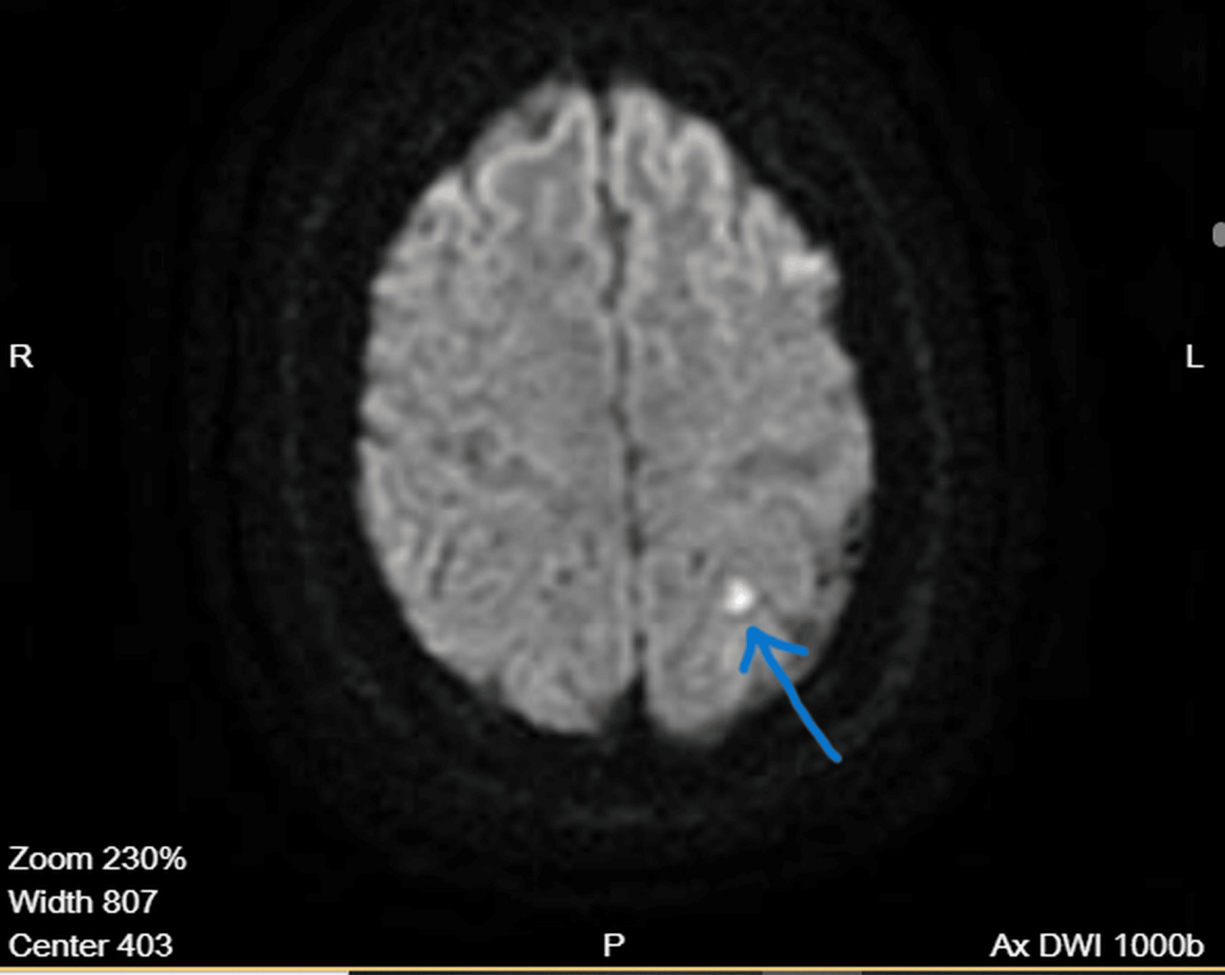
Apparent diffusion coefficient (ADC) map The corresponding apparent diffusion coefficient (ADC) map shows low signal intensity at the same location. The arrow highlights true diffusion restriction, supporting acute cortical ischemia.

MRI revealed two small punctate cortical foci of diffusion restriction within the left parietal lobe, corresponding to the distal M4 cortical territory of the left middle cerebral artery (Figure 1). These lesions demonstrated low apparent diffusion coefficient (ADC) values, consistent with acute ischemic infarction (Figure 2). No evidence of intracranial hemorrhage was identified.

**Figure 3:**
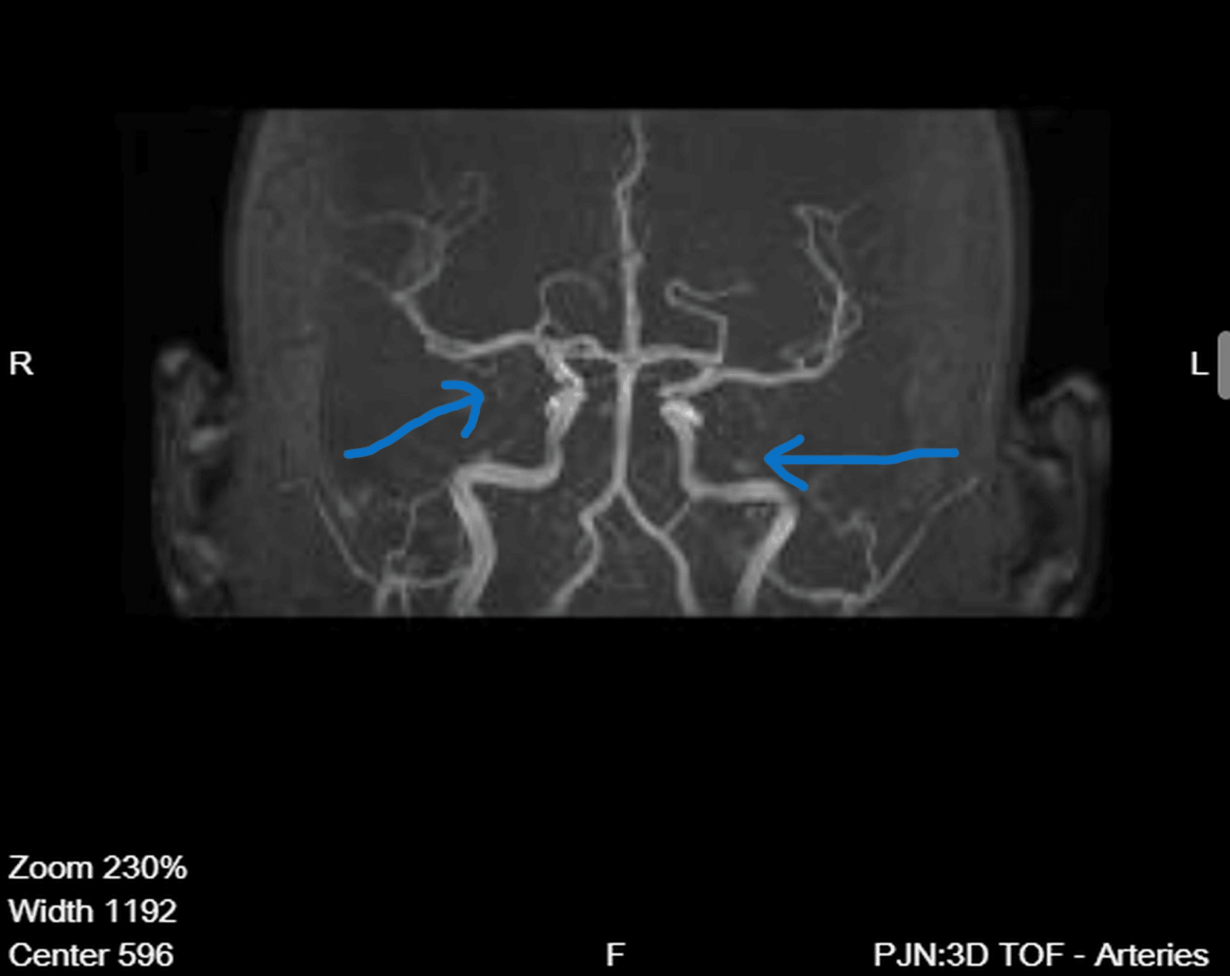
Magnetic resonance angiography-anterior view Three-dimensional time-of-flight magnetic resonance angiography (anterior view) demonstrates preserved flow within the Circle of Willis. The arrows indicate patent intracranial arteries without large-vessel occlusion.

**Figure 4:**
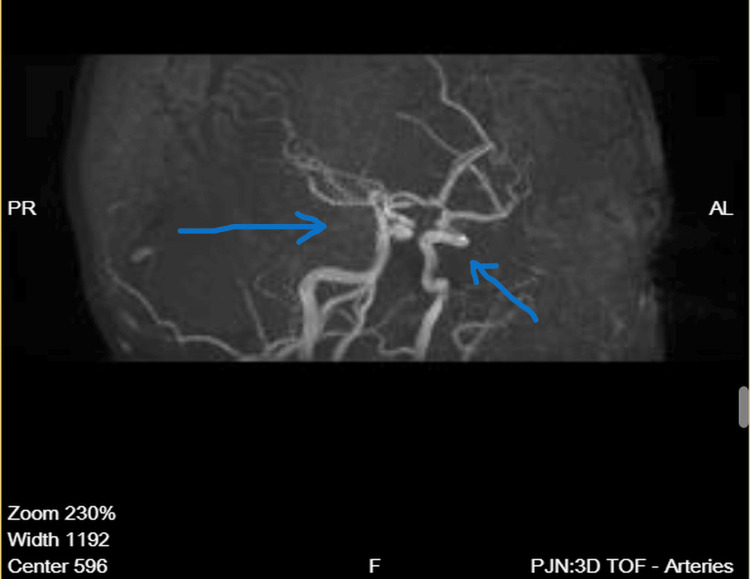
Magnetic resonance angiography – lateral view Lateral-view magnetic resonance angiography confirms intact flow within the intracranial arterial circulation. The arrows highlight normal-caliber cortical branches of the middle cerebral artery, with no vascular deformity, stenosis, or extrinsic compression identified.

Magnetic resonance angiography demonstrated preserved and symmetrical flow within the middle cerebral arteries, basilar artery, vertebral arteries, and the vessels of the Circle of Willis, without evidence of large-vessel occlusion, hemodynamically significant stenosis, vascular malformation, or extravascular compression (Figure 3). Flow within distal cortical branches appeared intact (Figure 4).

Laboratory investigations obtained on admission as part of the acute stroke evaluation were largely unremarkable. Coagulation studies, inflammatory markers, and routine metabolic panels were within normal limits. Thyroid function testing revealed mild subclinical hypothyroidism, and lipid analysis demonstrated borderline dyslipidemia (Table 1). There was no laboratory evidence of systemic infection, autoimmune disease, or an underlying hypercoagulable state.

**Table 1:** Summary of laboratory findings and reference ranges Laboratory evaluation revealed mild hypothyroidism and borderline dyslipidemia. Thyroid-stimulating hormone (TSH) was mildly elevated above the reference range, while lipid profile analysis demonstrated reduced high-density lipoprotein cholesterol (HDL-C) and mildly elevated low-density lipoprotein cholesterol (LDL-C). Other laboratory parameters were within normal limits.

Laboratory Test	Result	Reference Range
Thyroid-stimulating hormone (TSH)	5.182 µIU/mL	0.400–4.049 µIU/mL
HDL cholesterol (HDL-C)	1.25 mmol/L	>1.55 mmol/L
LDL cholesterol (LDL-C)	2.80 mmol/L	<2.59 mmol/L

Given the absence of traditional vascular risk factors, preserved large-vessel patency, and the distal cortical distribution of ischemic lesions, the stroke etiology was considered multifactorial. Chronic exposure to vasoconstrictive migraine therapy, in combination with intermittent NSAID use, minor metabolic abnormalities, and intermittent electronic cigarette exposure, was considered a potential contributing mechanism. The patient was initiated on appropriate secondary stroke prevention measures, and modifications to her migraine management were recommended, including reduction of vasoconstrictive pharmacotherapy.

During hospitalization, the patient’s neurological deficits gradually improved. At follow-up evaluation, she demonstrated marked clinical recovery with significant improvement in speech and motor function, and no new neurological deficits were identified.

## Discussion

In our patient, the absence of traditional cardiovascular risk factors raised concern for alternative mechanisms contributing to acute ischemic stroke, particularly given her young age and cortical infarct distribution. Migraine has been increasingly recognized as an independent risk factor for ischemic stroke, especially in young women, even in the absence of classic vascular comorbidities [5,17]. This association is thought to be multifactorial, involving transient cerebral hypoperfusion, endothelial dysfunction, platelet activation, and alterations in vascular reactivity [7].

Triptans, including sumatriptan, exert their therapeutic effect through selective agonism of serotonin 5-HT1B/1D receptors, resulting in cranial vasoconstriction and inhibition of neurogenic inflammation [9]. While generally considered safe when used intermittently and according to clinical guidelines, chronic or excessive triptan exposure has been postulated to be associated with ischemic events through sustained vasoconstriction, particularly in distal cortical vessels [10,11]. Case reports and pharmacovigilance data have described reversible cerebral vasoconstriction syndrome, transient ischemic attacks, and, more rarely, ischemic stroke in association with triptan use, although a definitive causal relationship remains unproven [12].

More recent literature has provided additional insight into triptan-associated cerebrovascular risk. A Danish case-crossover study involving over 429,000 triptan users reported that triptan initiation was associated with increased odds of ischemic stroke; however, the absolute risk was very low, estimated at approximately 1 event per 30,000 users [18]. Importantly, individuals who experienced cardiovascular events were generally older and had higher baseline cardiovascular risk, suggesting that triptan-related risk is not uniform across all users. In contrast, population-based cohort studies have demonstrated no increased cardiovascular risk in low-risk populations, and some analyses have even suggested a lower event rate among older adults using triptans compared with other migraine therapies [10,11].

In addition to triptan therapy, certain NSAIDs, including ketoprofen lysine salt (Fastenal) used by this patient, have been associated with cardiovascular and cerebrovascular risk. Proposed mechanisms include impaired endothelial prostaglandin synthesis, enhanced platelet aggregation, and effects on systemic blood pressure regulation [13,14]. In susceptible individuals, the combined use of vasoconstrictive agents and NSAIDs may synergistically lower the threshold for ischemic injury, particularly in the context of migraine-related vascular vulnerability. Certain NSAIDs have demonstrated dose-dependent associations with ischemic stroke risk, especially with prolonged use [15]. The patient also reported electronic cigarette use, which represents an emerging vascular risk factor. Experimental evidence suggests that electronic cigarette exposure is associated with endothelial dysfunction and impaired cerebral microvascular cell function mediated by endothelial-derived extracellular vesicles, resulting in reduced vasomotor reactivity an independent predictor of ischemic stroke which may further augment cerebrovascular vulnerability when combined with chronic vasoconstrictive migraine therapies [19].

The ischemic lesions observed in this patient were localized to the distal M4 cortical territory of the middle cerebral artery and were punctate in nature, a pattern consistent with small-vessel or distal cortical ischemia. Such lesions may be overlooked on non-contrast computed tomography, underscoring the diagnostic importance of magnetic resonance imaging in young patients presenting with acute focal neurological deficits [13].

Minor metabolic abnormalities, including mildly elevated thyroid-stimulating hormone levels consistent with subclinical hypothyroidism and borderline dyslipidemia, were also identified (Table 1). While unlikely to be independently causative, these factors may contribute to endothelial dysfunction and altered lipid metabolism, potentially augmenting cerebrovascular vulnerability when combined with other triggers [17].

Nevertheless, this case aligns with growing evidence that ischemic stroke in young adults often arises from complex, multifactorial interactions rather than a single causative factor [17,20]. A unique clinical insight from this case is that long-term daily triptan use combined with intermittent NSAID exposure may represent an underrecognized association with distal cortical ischemia in otherwise low-risk young adults, reinforcing the importance of careful medication history and individualized risk assessment. A detailed review of vasoconstrictive pharmacotherapy should therefore be an integral component of stroke evaluation in young patients with migraine.

## Conclusions

This case highlights that acute cortical ischemic stroke can occur in young adults without traditional cardiovascular risk factors and underscores a possible association between chronic migraine pharmacotherapy and cerebrovascular events. The combination of long-term daily triptan use, intermittent NSAID exposure, minor metabolic abnormalities, and concomitant electronic cigarette exposure may represent a synergistic contribution to distal cortical ischemia in otherwise low-risk individuals. Diffusion-weighted MRI proved critical for detecting small cortical infarctions that may be missed on computed tomography, with important implications for stroke evaluation in young patients with atypical presentations.

While the absolute risk of ischemic stroke in young migraine patients remains low and causality cannot be established from a single case, this presentation reinforces the importance of heightened clinical vigilance in patients with prolonged exposure to vasoconstrictive migraine therapies, particularly when multiple agents or additional vascular risk modifiers are present. Consideration of individualized cerebrovascular risk assessment may be appropriate in selected patients requiring long-term daily triptan therapy. Further prospective studies are needed to better characterize cerebrovascular risk and inform evidence-based safety guidance for chronic migraine pharmacotherapy.
